# Disentangling a metabolic cross-feeding in a halophilic archaea-bacteria consortium

**DOI:** 10.3389/fmicb.2023.1276438

**Published:** 2023-12-21

**Authors:** Nahui Olin Medina-Chávez, Abigail Torres-Cerda, Jeremy M. Chacón, William R. Harcombe, Susana De la Torre-Zavala, Michael Travisano

**Affiliations:** ^1^Department of Ecology, Evolution and Behavior, University of Minnesota, St. Paul, MN, United States; ^2^BioTechnology Institute, University of Minnesota, St. Paul, MN, United States; ^3^Universidad Autónoma de Nuevo León, Facultad de Ciencias Biológicas, Instituto de Biotecnología, San Nicolás de los Garza, San Nicolás de los Garza, Mexico; ^4^Minnesota Supercomputing Institute, Minneapolis, MN, United States; ^5^Minnesota Center for the Philosophy of Science, University of Minnesota, Minneapolis, MN, United States

**Keywords:** syntrophy, cross-feeding, metabolic exchange, archaea, bacteria, halophiles, extremophiles

## Abstract

Microbial syntrophy, a cooperative metabolic interaction among prokaryotes, serves a critical role in shaping communities, due to the auxotrophic nature of many microorganisms. Syntrophy played a key role in the evolution of life, including the hypothesized origin of eukaryotes. In a recent exploration of the microbial mats within the exceptional and uniquely extreme Cuatro Cienegas Basin (CCB), a halophilic isolate, designated as AD140, emerged as a standout due to its distinct growth pattern. Subsequent genome sequencing revealed AD140 to be a co-culture of a halophilic archaeon from the *Halorubrum* genus and a marine halophilic bacterium, *Marinococcus luteus*, both occupying the same ecological niche. This intriguing coexistence hints at an early-stage symbiotic relationship that thrives on adaptability. By delving into their metabolic interdependence through genomic analysis, this study aims to uncover shared characteristics that enhance their symbiotic association, offering insights into the evolution of halophilic microorganisms and their remarkable adaptations to high-salinity environments.

## Introduction

Syntrophy, or cross-feeding, is widely distributed and occurs when a microbial strain consumes substrates produced by other microbial strains, promoting the growth of the cross-fed strain (Escalante et al., 2015). It is central in structuring microbial communities because most microorganisms are auxotrophic (Zengler and Zaramela, 2018), and cross-fed substrates provide essential resources that would be otherwise unavailable. Without syntrophic interactions, most microbial communities would collapse to relatively few prototrophic species. Syntrophic interactions were central in the evolutionary history of life, as eukaryotes are hypothesized to have evolved from a mutually beneficial syntrophy between an archaeon and a bacterium. Archaea and Bacteria are highly divergent, having long phylogenetically distinct evolutionary histories, and archaea are thought to be especially dependent upon syntrophic interactions (López-García and Moreira, 2019; López-García and Moreira, 2020). Even so, species from both domains share many features, size, morphology, movement, and defense mechanisms and were long thought to be one taxonomic group (Medina-Chávez and Travisano, 2022). Disentangling syntrophies between archaea and bacteria will shed light on current microbial communities and potentially on the evolutionary origins of eukaryotes.

Recent research has unveiled the remarkable diversity within microbial mats from the Cuatro Cienegas Basin (CCB) (Espinosa-Asuar et al., 2022), where similar to other extreme environments, the presence of archaea and extremophilic bacteria is prominent. CCB is an oasis in the Chihuahuan Desert in Coahuila, Mexico, characterized by its oligotrophic states, which involve phosphorus depletion, high salinity, high pH, and high radiation levels (Souza et al., 2008; Medina-Chávez et al., 2020). Within CCB, a plethora of microbial diversity has been described in previous reports (Bonilla-Rosso et al., 2012; Peimbert et al., 2012; Arocha-Garza et al., 2017). Due to its geographic isolation, species have evolved without interruption, resulting in a long and stable period of metabolic compatibility and adaptation for community assembly (Souza et al., 2018). Notably, one halophilic isolate designated as AD140 exhibits a distinctive growth pattern on agar plates. First, yellow-colored colonies grow over a two-week span, followed by red colonies 4 weeks after the initial striking. This is unique since the red colonies do not grow on top of the agar media but rather directly on top the yellow biomass. This is in contrast to other isolates in co-culture where they do not grow in physical contact with each other. Through genome sequencing, strain AD140 was identified as a co-culture between a halophilic archaeon from the *Halorubrum* genus (red colonies) and a marine halophilic bacterium, *Marinococcus luteus* (yellow colonies). Despite numerous attempts over the years to separate both strains in axenic cultures, with the use of antibiotics, serial-dilution to extinction, spent media, different media composition, varying ph or temperature, neither microorganism thrive in isolation. These two lineages occupy the same abiotic ecological niche and have developed comparable mechanisms of adaptation and stress response. In addition to thriving in salt-saturated conditions, they demonstrate resilience during periods of desiccation and fluctuations in other environmental factors (Medina-Chávez et al., 2023). The co-occurrence of these two strains as a sufficient symbiont interaction in laboratory settings led us to propose it as an early-stage symbiosis, where their symbiotic continuum depends on their adaptability in an eco-evolutionary framework.

The objective of this study was to explore the genomic features of a halophilic archaea and marine bacteria consortium through comparative genomic analysis, with the aim of understanding the metabolic co-dependence that shapes the early stages of symbiosis. Symbiosis through metabolic cross-feeding is enhanced by genome features such as genomic plasticity, horizontal gene transfer events, symbiosis-specific genes, and regulatory mechanisms, leading to changes in the organization of functional elements in the genome (Hentschel et al., 2000; Ochman and Moran, 2001; Moran, 2007; Juhas et al., 2009). Through comprehensive genomic analysis and metabolic network prediction, we describe the characteristics of these two microorganisms and their close interaction based on a set of shared traits. These findings have the potential to illuminate the convergent evolution of halophilic microorganisms and provide insights into the genetic basis of their adaptations to high-salinity environments.

## Materials and methods

### Sampling

Environmental sampling of water and sediment was collected in Cuatro Cienegas, Coahuila (26° 49′ 41.7″ N, 102° 01′ 28.7″ O), with ~10 cm of depth in sterile Falcon tubes.

### Culture conditions

For the first stage of culture (primary isolation) AM2 culture media was designed and used. AM2 contains ([Per liter]): 21.2 g bacteriological agar, 2.4 g dextrose, 5 g peptone (Oxoid), 5 g yeast extract, 3 g Na_3_C_6_H_5_O_7_, 0.2 g CaCl_2_ 2H_2_O, 50 g MgCl_2_ 6H_2_O, 20 g MgSO_4_ 7H_2_O, 2 g KCl, 250 g NaCl. For the second stage of isolation, ATCC 213 media was used ([Per liter]): 25 g bacteriological agar, 3.1 g tryptone, 12.5 g yeast extract, 0.4 g CaCl_2_ 2H_2_O, 50 g MgCl_2_ 6H_2_O, 12.5 g MgSO_4_ 7H_2_O, 6.2 g KCl, 250 g NaCl. Both media were adjusted at pH = 7.4. Liquid and agar media were used, and incubation was held at 28°C.

### Growth curves

Samples of 1 mL from the cultures were extracted aseptically from the tubes to assess growth via optical density (O.D.) measurements at 600 nm using an Agilent Technologies Cary 100 UV–Visible Model G9821A spectrophotometer. This experiment was replicated three times for accuracy.

### Antibiotic and biofilm test

To assess the interdependency between archaea and bacteria, we conducted an antibiotic susceptibility test. We initiated the test by inoculating 100 μL of co-culture into 10 glass tubes with 6 mL of 213 media and 6 μL of ampicillin (final concentration 50 mg/mL, Roche), active against *M. luteus*, but not *Halorubrum* sp. followed by incubation at 28° C, 250 rpm, during 5 weeks. For positive controls we maintained another 10 tubes of 213 media without ampicillin and 100 μL of AD140 culture, as negative controls we used five media tubes with no antibiotic and without inoculum.

To test for biofilm production, we grew the co-culture at 28° C, using 6 mL of liquid 213 media and a 200 μL inoculum, which corresponds of approximately 5 × 10^6^ cells, until full culture growth. We did not used the standard 48 or 96-well assay plate due to the large amount of salt in the media, the long period of static state and the small volume commonly used in well-assay plates, leading to evaporation of the media and precipitation of salts.

### Genomic DNA extraction and whole genome sequencing

To identify the isolated strains at the species taxonomic level, genomic DNA extraction was performed. Phenol-Chloroform (1:1) protocol was used with the next modifications: biomass pellet was obtained from agar plates and resuspended in 400 μL phenol-chloroform solution and 400 μL of QTP lysis buffer which contained [Per liter] (40 mL Triton X-100, 100 mL SDS 20%, 20 mL NaCl 5 M, 10 mL Tris 2 M pH = 8,7 mL EDTA pH = 8, and milliQ water was added up to 10 mL), into 2 mL Eppendorf tubes with 0.2 μL of glass beads (0.1 mm Ø). The tubes were shaken in a rotational disruptor (MagNA Lyser Instrument, Roche Diagnostics) for 1 min at 5,600 rpm and incubated for 1 min in ice (4° C, 3× times). For DNA precipitation, the liquid phase was taken and placed into a new 2 mL Eppendorf tube adding 25 μL sodium acetate, 95% ethanol to the top, and incubated at −20° C overnight. The tubes were centrifugated recovering the pellet and washing it twice with 70% ethanol. DNA was resuspended in 30 μL of TE buffer + RNAse and stored at −20° C. Sequencing was done by CINVESTAV-LANGEBIO Irapuato, Mexico, using Illumina MiSeq technology 2 × 300 with a 150X depth.

### Assembly and binning

Read quality control was performed with FastQC v0.11.9 (Andrews, 2010). The reads were cleaned and trimmed using Trimmomatic v0.33 (Bolger et al., 2014) using the parameters as it follows: *trimmomatic-PE-phred33 input_forward.fq.gz input_reverse.fq.gz output_forward_paired.fq.gz output_forward_unpaired.fq.gz output_reverse_paired.fq.gz output_reverse_unpaired.fq.gz ILLUMINACLIP:TruSeq3-PE.fa:2:30:10 LEADING:3 TRAILING:3 SLIDINGWINDOW:4:15 MINLEN:36*. Once cleaned and trimmed, the sequences were assembled *de novo* with MetaSpades v.3.15.5 (Nurk et al., 2017) using 21, 31, 51, 61, and 71 k-mers. The quality analysis of the assembly was performed using MetaQUAST v5.2.0 (Mikheenko et al., 2015).

To retrieve individual bins, the assembly obtained with MetaSpades was analyzed with MaxBin2 v2.2.7 (Wu et al., 2014). To make sure it was not a contamination, we check genome quality using CheckM v.2 using default parameters. Genomic fasta sequences and abundance values from *Halorubrum* sp. and *Marinococcus luteus* were retrieved.

### Annotation

Genomes of *Halorubrum* sp. and *Marinococcus luteus* were annotated using Prokka v1.14.6 software (Seemann, 2014), establishing in the parameters the use of an archaea genome data bank from RefSeq, NCBI (see full annotation in Supplementary Tables 8, 9). Also, RASTtkv1.3.0 was used for additional annotation (Brettin et al., 2015).

### Biosynthetic clusters and genomic island prediction

To predict biosynthetic clusters, we used antiSMASH 7.0 with default parameters as it follows: antismash --cb-general --cb-knownclusters --cb-subclusters --asf --pfam2go --smcog-trees --tigrfam --output-dir antismash/prokka_06092021.merged.gbk.Genomic islands prediction was performed using GYPSy, and AlienHunter. GYPSy algorithm compares the genome of interest with a reference genome, considering only the regions that are absent in the reference genome; as for Alien hunter, it employs the Interpolated Variable Order Motifs (IVOMs) method to forecast potential HGT regions based solely on sequence composition, without needing a reference genome.

### KEGG analysis

The protein sequences retrieved from Prokka were analyzed with BlastKOALA (Kanehisa et al., 2016) to obtain the KEGG Orthology assignation (KO) of each gene. The KO output was introduced into KEGG Mapper Reconstruct (Kanehisa et al., 2022) to annotate the enzymes and metabolic pathways from both genomes.

### CarveMe analysis

Individual genome-scale metabolic models were generated using the CarveMe pipeline (Machado et al., 2018), using the .fasta files of the protein sequences obtained from Prokka annotation and media composition used in the culture.

### Flux balance analysis

Metabolic requirements of each genome-scale metabolic model were determined by applying flux-balance analysis using the python tool COBRApy (Ebrahim et al., 2013). To determine basic needs, each model was initially provided with all nutrients the model is capable of taking up. Then each nutrient was removed one-at-a-time, and nutrients whose removal prevented growth were deemed essential.

To predict potential minimal media, we used the “minimal_medium” function in COBRApy, set to minimize the number of nutrients required (as opposed to minimizing total uptake flux). We did the 1e6 times to try to find all possible minimal media (i.e., we used the argument minimize_components = 1,000,000).

To test whether each model had the potential to synthesize the nutrients the other model required environmentally, we used flux balance analysis with production of the metabolite in question as the objective function. Specifically, a model was provided with one of the media identified in the minimal media analysis. Then, a sink reaction was added to the model which could draw one specific metabolite out of the intracellular space. This was done one-at-a-time for each non-ion needed by the other species. Then, flux-balance analysis was used with the sink reaction as the objective function. If the solution >1e-6, we state that the model could produce this nutrient.

### Data availability

Complete genome data is available at NCBI BioProject PRJNA1003106.

## Results

### Phenotypic description of AD140 consortium

**Figure 1:**
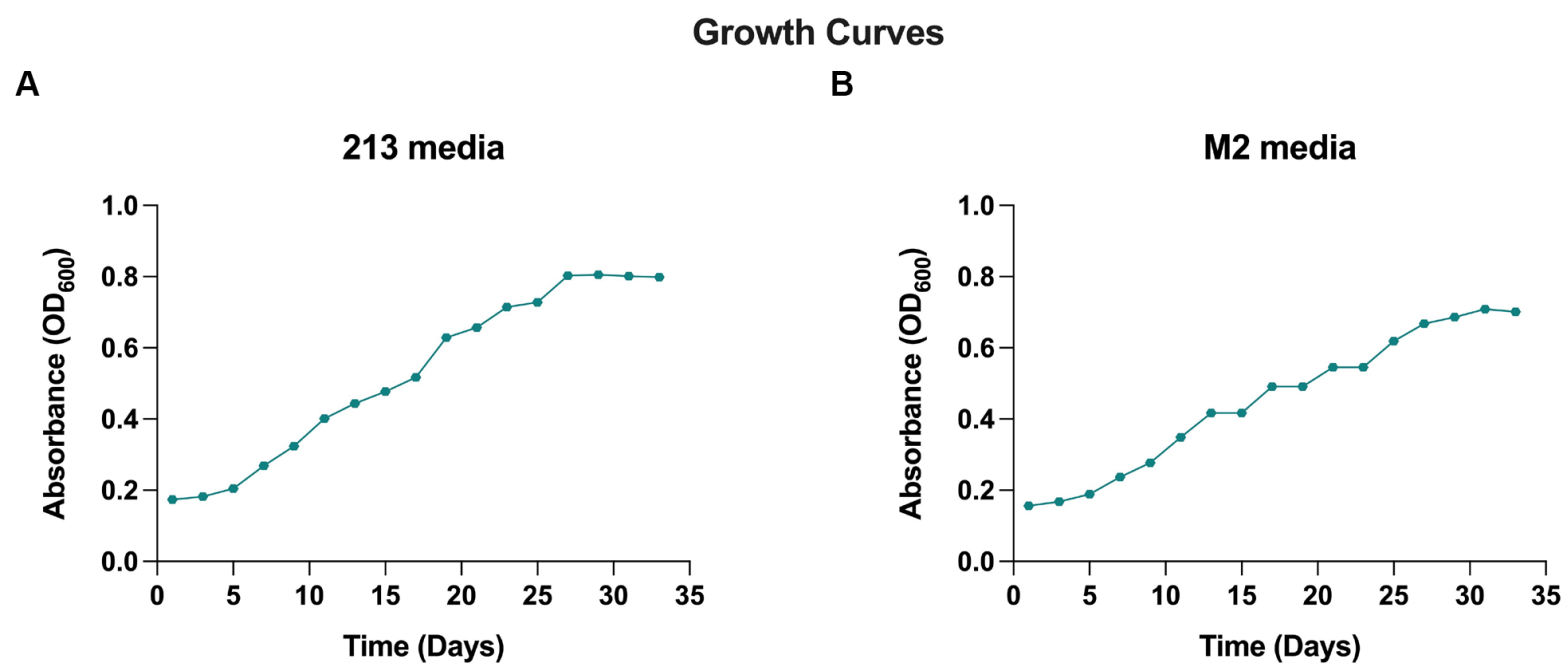
Growth curve of AD140 co-culture, in **(A)** 213 media and **(B)** M2 media (25% Salinity, temperature 28°C, pH = 7.4).

**Figure 2:**
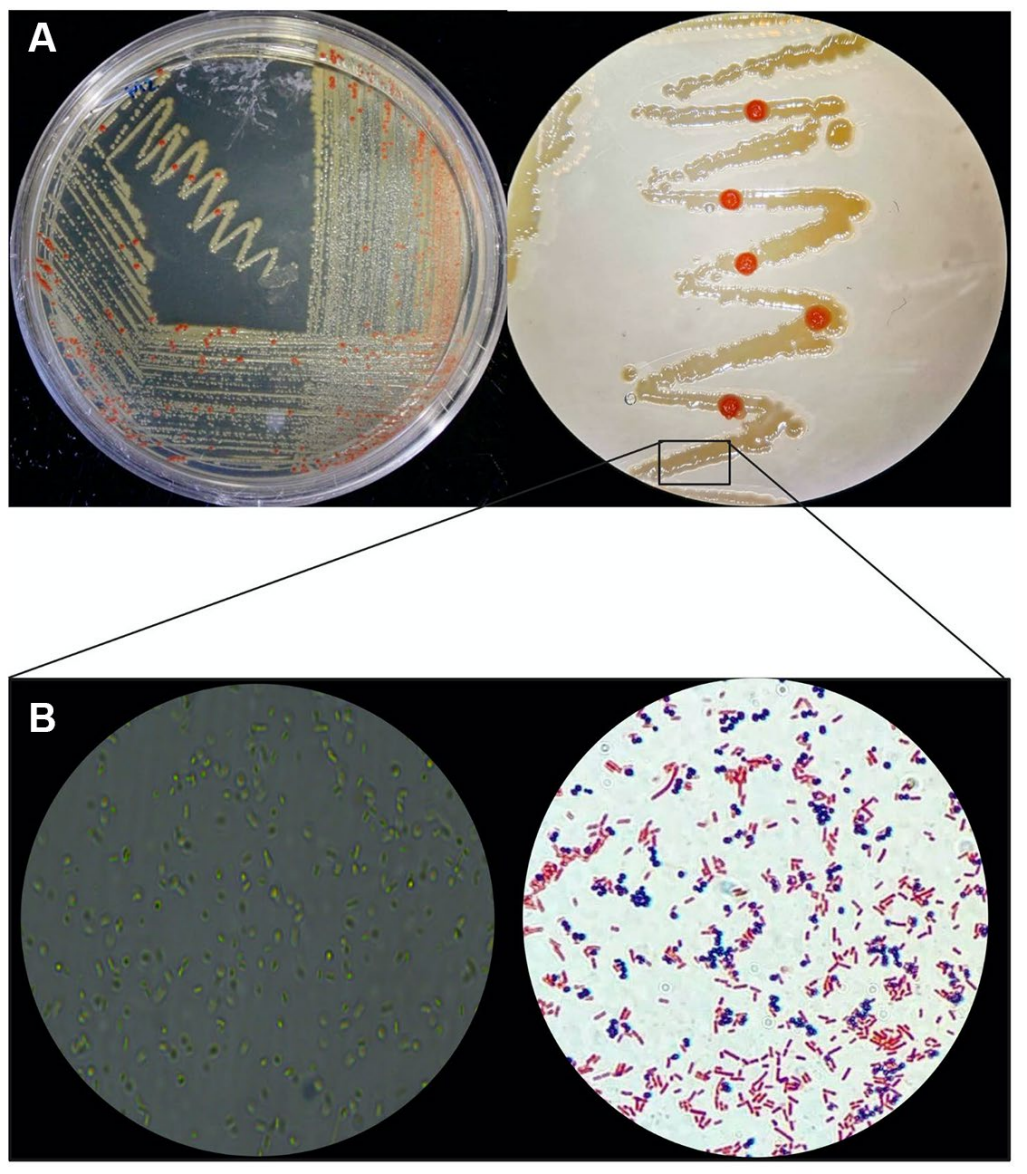
Phenotypic macro and microscopic characterization of AD140 co-culture. **(A)** *Halorubrum* sp. (red colonies) and *Marinococcus luteus* (yellow colonies) distinctive growing pattern on 213 media plates. **(B)** Microscopy and gram stain of the consortium (Eclipse Ci Microscope, 100× magnification), taken from a smear of the yellow biomass showing gram-negative rods and gram-positive cocci belonging to *Halorubrum* sp. and *Marinococcus luteus*, respectively. Created with BioRender.com.

When cultivated in liquid media, it exhibits rapid growth at 28°C with agitation at 250 rpm, in 18–20 days. The AD140 consortium shows optimal growth in 213 and M2 media, reaching a maximum at day 29 and 31, respectively, after which it entered stationary phase (Figure 1). On agar plates, circular colonies with a yellow color begin to emerge after 14 days, subsequently identified as the bacterium, *Marinococcus luteus*. Over the course of 25–28 days, circular colonies with a red color appear atop the initial yellow colonies, which are identified as the archaeon, *Halorubrum* sp. (Figure 2).

**Figure 3:**
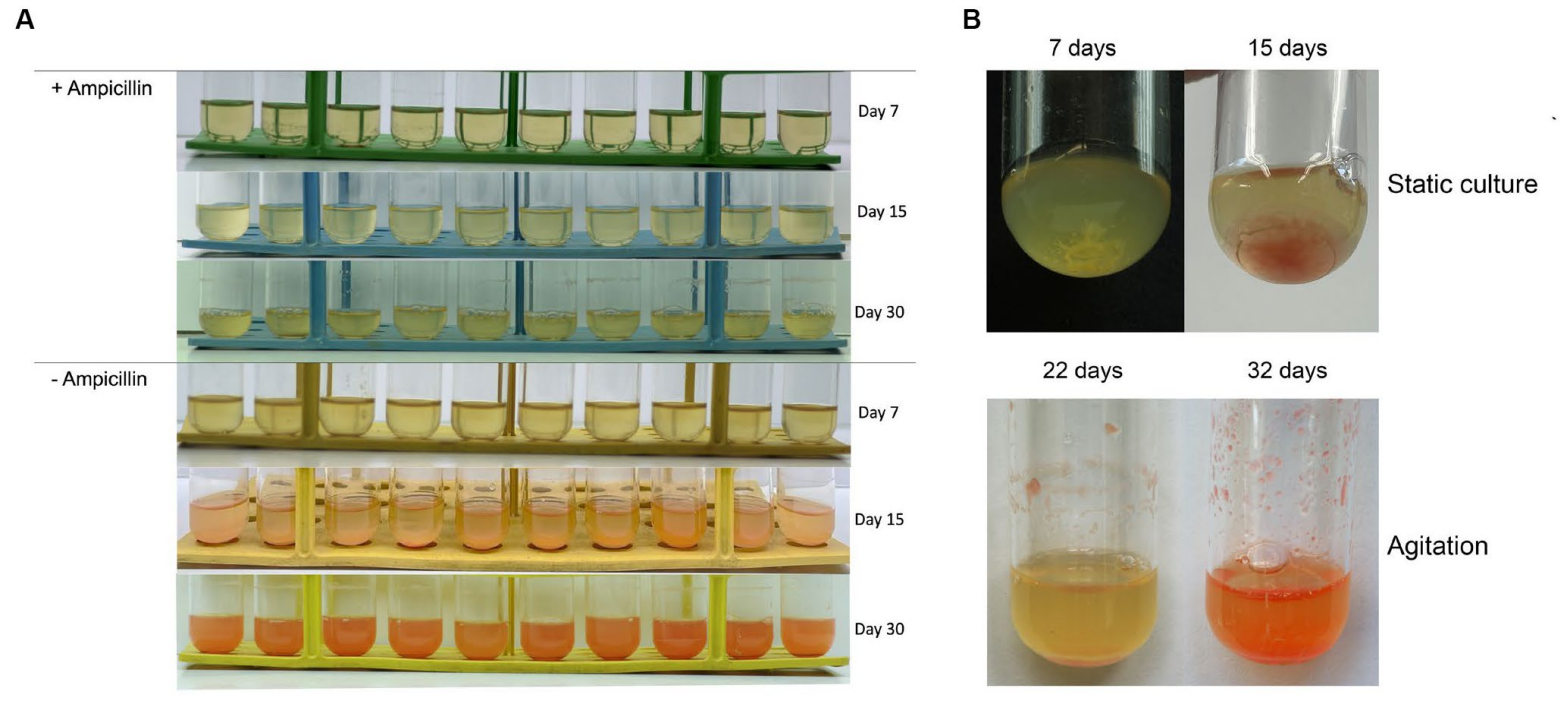
**(A)** Tubes containing ampicillin (1 μg/mL) show no growth to minimum up until 15 days and continue to decline afterwards. *Halorubrum* sp., does not grow while *Marinococcus luteus* is eliminated from the culture. While the tubes with no antibiotic developed as expected. **(B)** Biofilm formation was observed at the bottom of the culture tube during the first 15 day of culture under static conditions, meanwhile aggregation and attachment of the biofilm was observed from day 22 to day 32 under agitation regime.

To validate the mutualistic relationship, an antibiotic test was conducted making sure to target one of the microorganisms. The distinctive variances in the cell walls of archaea and bacteria allow for selective susceptibility to antibiotics. Ampicillin, a beta-lactam antibiotic, is widely used against gram-positive bacteria and has no effect on archaea. In the tubes designated for antibiotic exposure, either no visible growth or minimal growth was observed, declining afterwards. The absence of visible growth or minimal growth in these tubes indicates that the antibiotics tested successfully inhibited the proliferation of *Marinococcus luteus*. This outcome suggests that the microorganism is sensitive to the antibiotics used in the assay, as they effectively suppressed its growth or completely prevented its replication, but more importantly, *Halorubrum* sp. did not grow when the bacteria was eliminated from the culture. This shows that the propagation of *Halorubrum* is dependent from *Marinococcus* (Figure 3).

### Genome identification and characterization of AD140 consortium

Whole genome sequencing was performed to identify the halophilic isolate. The number of raw reads obtained after *pair-end* sequencing was 4,489,067 reads. The first assembly draft of the AD140 isolate obtained from Spades, resulted in a 7,796,468 bp genome with 2,729 contigs. The GC% average was found to be 58.81%, and the functional annotation revealed 7,860 coding genes, 147 tRNAs and 9 rRNAs. Compared to other haloarchaea genome sizes, we observed that the larger genome size of the AD140 genome was not typical for this taxon. The significant increase of ~2,310,000 bp, the abundance of genes, and the unique phenotypic growth led us to hypothesize that the AD140 culture could hold more than one specie. To confirm the presence of several microorganisms in the same sample, we performed assembly using MetaSpades and subsequently utilized MaxBin for automatic binning. The process yielded two distinct genomes, confirming the identity of *Halorubrum* sp. an archaeon, and *Marinococcus luteus* a bacterium. The presence of both members was supported by genome quality check, showing the Archaea genome had a completeness of 99.07% and contamination ratio of 4.48, while the Bacteria, showed a completeness 98.28% and contamination ratio of 0. The genomic features of both genomes are presented in Table 1.

**Table 1:** Genomic features of *Halorubrum* sp. AD140 and *Marinococcus luteus* AD140.

Genomic features		
	*Halorubrum* sp. AD140	*Marinococcus luteus* AD140
Size (bp)	3,552,188	2,976,466
Contigs	1,061	64
Contigs > 500 pb	711	24
Longest contig size (bp)	107,395	464,553
N50	30,273	319,712
GC%	68.98%	47.77%
CDS	3,556	3,021
tRNA	68	78
rRNA	4	4

### Functional annotation

**Figure 4:**
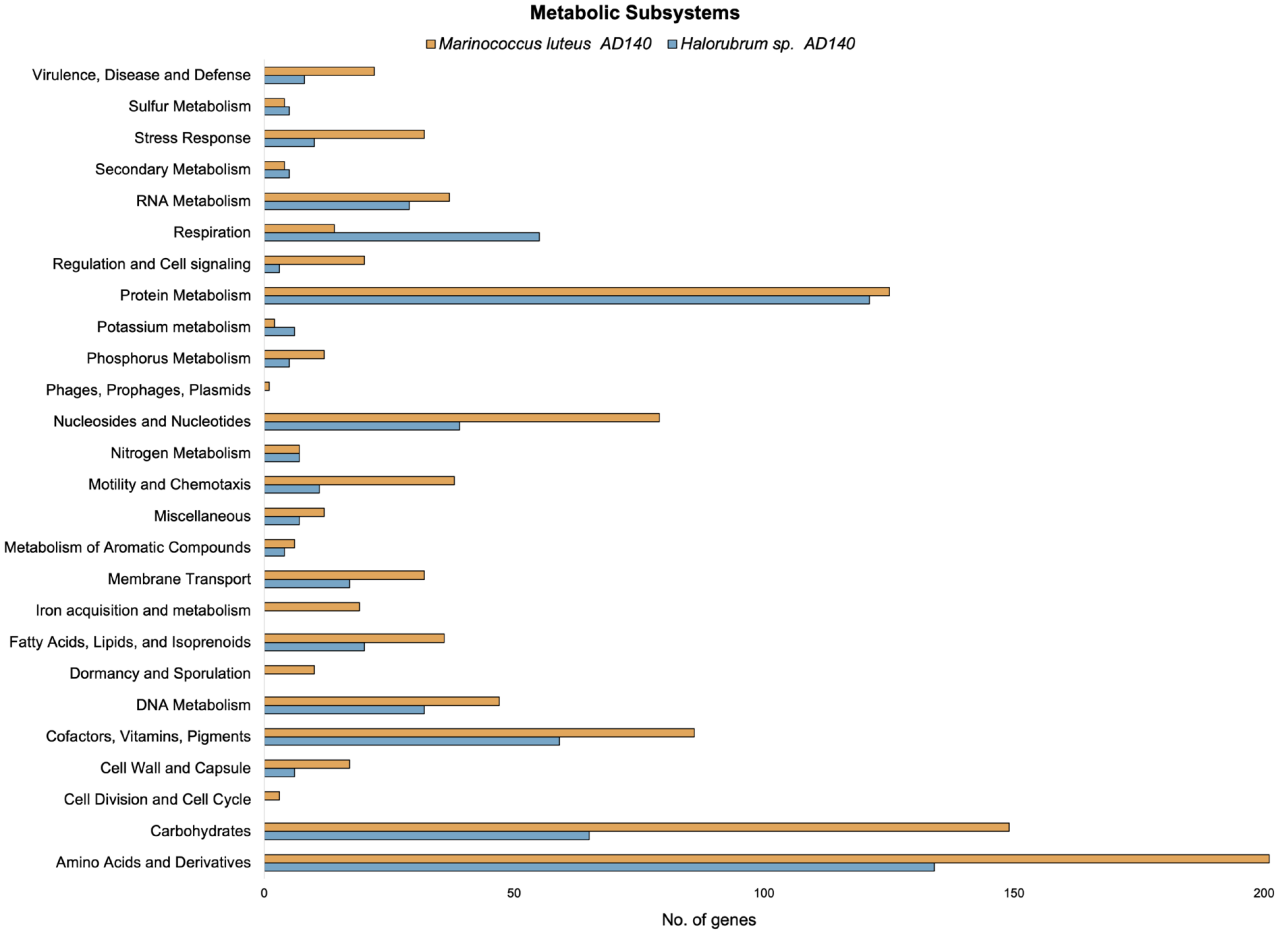
Comparison of the number of genes in different metabolic subsystems based on RAST annotation of *Marinococcus luteus* (yellow) and *Halorubrum* sp. (blue).

Preliminary functional annotation was conducted using the RAST Server: Rapid Annotation using Subsystems Technology (Brettin et al., 2015). The annotation process involved the identification of protein-encoding, rRNA and tRNA genes through homology, and assigning these proteins to specific metabolic subsystems (Figure 4). The construction of these models was performed for both genomes. Complete functional annotation using prokka v1.14.6, allow to explore gene regulatory systems and transposable elements associated with Horizontal Gene Transfer (Table 2).

**Table 2:** Features related to Horizontal Gene Transfer (HGT) in *Halorubrum* sp. AD140 and *Marinococcus luteus* AD140.

Features related to horizontal gene transfer (HGT)		
	*Halorubrum* sp.	*Marinococcus luteus*
Genomic Islands	30	13
Transposase genes	21	17
CRISPR elements	0	0
Biosynthetic gene clusters	1	3
Biofilm genes	47	131

### Genomic islands in AD140 co-culture

A comprehensive analysis revealed the presence of 30 genomic islands within the genome of *Halorubrum* sp., while *M. luteus* exhibited 13 genomic islands. These islands were predominantly located in regions characterized by low %GC content, indicating their likely acquisition from other organisms. Notably, these islands harbored genes associated with the mobilization of genomic material, such as integrases, recombinases, transposases, and exonucleases. In the case of *Halorubrum* sp., mobile elements were specifically identified within islands 6, 8, 9, 18, 20, 22, and 29 (Table 3), whereas for *M. luteus*, were identified in islands 1, 4, 5, 6, 7, 9, 10, and 13 (Table 4). Complete genomic islands descriptions can be found in Supplementary Tables 3, 4, respectively.

**Table 3:** Description of Genomic Islands of *Halorubrum* sp. AD140.

Genomic Island	Start	End	Putative function	Prediction algorithm
1	41,767	62,852	Symbiotic	Gipsy
2	177,500	190,000	Unknown	Alien_Hunter, Gipsy
3	192,494	199,280	Resistance	Gipsy
4	206,799	214,549	Resistance, Symbiotic, Metabolic	Gipsy
5	396,667	404,724	Unknown	Gipsy
6	517,500	537,500	Unknown	Alien_Hunter, Gipsy
7	595,433	613,758	Unknown	Gipsy
8	656,604	663,702	Unknown	Alien_Hunter, Gipsy
9	698,217	726,086	Unknown	Alien_Hunter, Gipsy
10	735,000	745,000	Resistance, Metabolic	Alien_Hunter, Gipsy
11	755,987	770,258	Metabolic, Symbiotic	Gipsy
12	820,550	827,502	Unknown	Gipsy
13	966,569	988,737	Pathogenicity, Resistance, Metabolic	Gipsy
14	992,464	1,007,785	Metabolic	Gipsy
15	1,108,805	1,120,239	Pathogenicity, Symbiotic	Gipsy
16	1,160,266	1,170,568	Unknown	Gipsy
17	1,215,000	1,255,000	Metabolic	Alien_Hunter, Gipsy
18	1,457,500	1,479,265	Unknown	Alien_Hunter, Gipsy
19	1,513,626	1,527,834	Metabolic, Pathogenicity, Resistance	Gipsy
20	1,845,000	1,852,500	Metabolic	Alien_Hunter
21	2,075,528	2,084,135	Unknown	Gipsy
22	2,114,057	2,140,000	Resistance	Alien_Hunter, Gipsy
23	2,183,397	2,197,236	Metabolic, Pathogenicity, Resistance, Symbiotic	Gipsy
24	2,257,723	2,277,500	Metabolic, Pathogenicity, Resistance	Alien_Hunter, Gipsy
25	2,294,475	2,314,987	Unknown	Gipsy
26	2,508,816	2,522,980	Unknown	Gipsy
27	2,617,771	2,623,524	Unknown	Gipsy
28	2,668,936	2,683,515	Unknown	Gipsy
29	2,790,000	2,797,500	Unknown	Alien_Hunter, Gipsy
30	2,847,491	2,854,584	Unknown	Gipsy

**Table 4:** Description of Genomic Islands of *Marinococcus luteus* sp. AD140.

Genomic Island	Start	End	Putative function	Prediction algorithm
1	268,740	298,778	Pathogenicity, Symbiotic	Alien_Hunter, Gipsy
2	308,483	320,869	Resistance	Gipsy
3	427,309	441,922	Resistance	Gipsy
4	1,285,000	1,342,500	Unknown	Alien_Hunter, Gipsy
5	1,380,000	1,405,000	Unknown	Alien_Hunter
6	1,593,859	1,610,000	Resistance	Alien_Hunter, Gipsy
7	1,877,089	1,900,000	Resistance	Alien_Hunter, Gipsy
8	1,907,905	1,920,470	Metabolic, Pathogenicity, Resistance, Symbiotic	Gipsy
9	2,077,690	2,092,500	Unknown	Alien_Hunter, Gipsy
10	2,154,096	2,171,409	Metabolic, Pathogenicity, Resistance, Symbiotic	Gipsy
11	2,471,823	2,482,818	Pathogenicity, Resistance, Symbiotic	Gipsy
12	2,834,662	2,840,700	Unknown	Alien_Hunter, Gipsy
13	2,962,500	2,977,500	Unknown	Alien_Hunter

Phage infection was identified as the probable mechanism of acquisition for specific islands, namely islands 6, 8, and 22 in *Halorubrum*, and island 4 in *M. luteus*, as these islands contained genes encoding phage proteins. Moreover, both microorganisms have genomic islands containing resistance genes. The archaeon, *Halorubrum* sp., possess islands associated with cadmium resistance (island 3) and arsenic resistance (island 24). Furthermore, island 13 harbors genes encoding metallo-beta-lactamase, while island 22 contains genes for multidrug resistance proteins. Similarly, to *Halorubrum* sp., *Marinococcus luteus,* has an island (island 7) carrying an arsenic resistance gene. Additionally, it exhibits genes related to fluoroquinolone resistance in island 2.

In *Halorubrum* sp., several islands containing genes involved in metabolite synthesis were identified. For instance, island 4 is associated with succinoglycan synthesis, island 14 with thiazole synthesis, and island 20 with cisthiatonine synthesis. Additionally, islands with metabolic features were discovered, including island 10 (associated with amilase production), island 13 (related to thiaminase activity) and island 17 (involved in sulfur metabolism) On the other hand, *M. luteus*, genomic islands with genes related to metal metabolism were also identified. Specifically, island 8 contains genes related to iron metabolism, as well as genes encoding nitrous oxidoreductase, an enzyme involved in the production of nitrogen (N_2_) from nitrous oxide (N_2_O).

### Biosynthetic clusters

**Figure 5:**
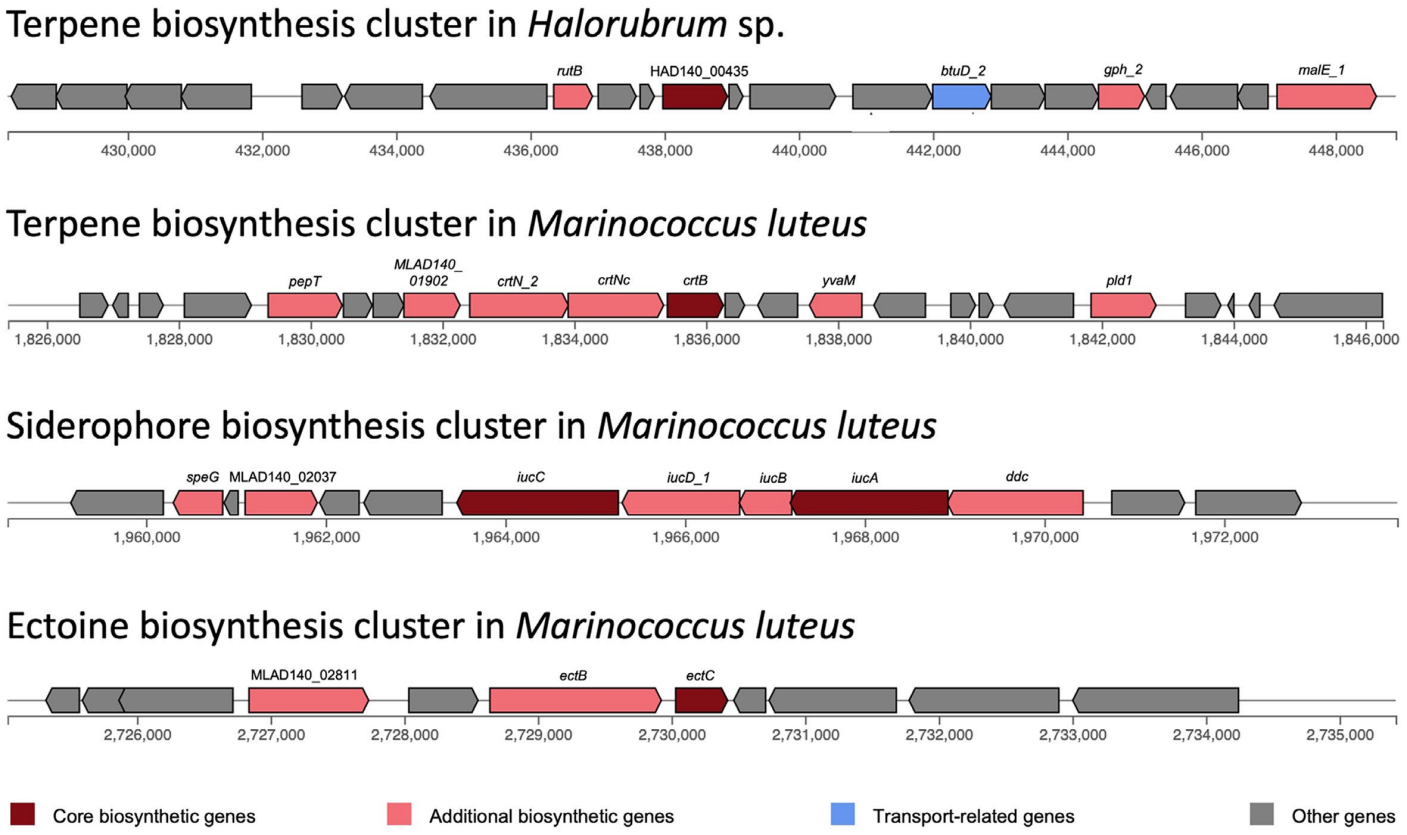
Biosynthesis clusters predicted in *Halorubrum* sp. and *Marinococcus luteus* AD140, using antiSMASH v6.0.0. Each block represents a gene.

Biosynthetic gene clusters were identified with antiSMASH. Only one biosynthetic cluster was found in *Halorubrum* sp., while three clusters were found in *Marinococcus luteus* (Figure 5). Both species have a terpene biosynthesis clusters, noteworthy, the bacterium terpene cluster did not have transport related genes, but several additional biosynthetic genes, with a very distinctive arrangement. The terpene biosynthesis cluster in *Halorubrum* sp. contains a phytoenesynthase gene (HAD140_00435), three additional biosynthetic genes: *rutB* (isochorismatase), *gph_2* (HAD hydrolase), and *malE_1* (sugar-binding lipoprotein), and one transport-related gene: *btuD_2* (phosphonate ABC transporter). While the terpene biosynthesis cluster in *Marinococcus luteus* also contains a phytoene synthase gene (*crtB*), the other genes in the cluster are different from the ones found in the terpene cluster in *Halorubrum* sp. The cluster in *M. luteus* does not have any transport genes and has six additional biosynthetic genes: *pepT*, MLAD140_01902, *crtN_2*, *crtNc*, *yvaM*, and *pld1*.

The siderophore biosynthesis cluster in *M. luteus* contains two core biosynthesis genes: *iucA* and *iucC*. This cluster also has five additional biosynthetic genes: *speG*, MLAD140_02037, *iucD_1*, *iucB* and *ddc*. Moreover, an ectoine biosynthesis cluster was identified in *M. luteus*. This cluster has one core biosynthetic gene (*ectC*) and two additional biosynthetic genes: *ectB* and MLAD140_02811.

### CRISPR-Cas elements

A total of 9 CRISPR elements were found in the genome of *Halorubrum* sp. using CRISPRCasFinder. Nevertheless, these elements were predicted with level 1 evidence, the lowest prediction level of CRISPRCasFinder. Complete features of predicted CRISPR elements are found in Supplementary Table 5. Meanwhile, *Marinococcus luteus* did not show any CRISPR element.

### Biofilm production genes

**Figure 6:**
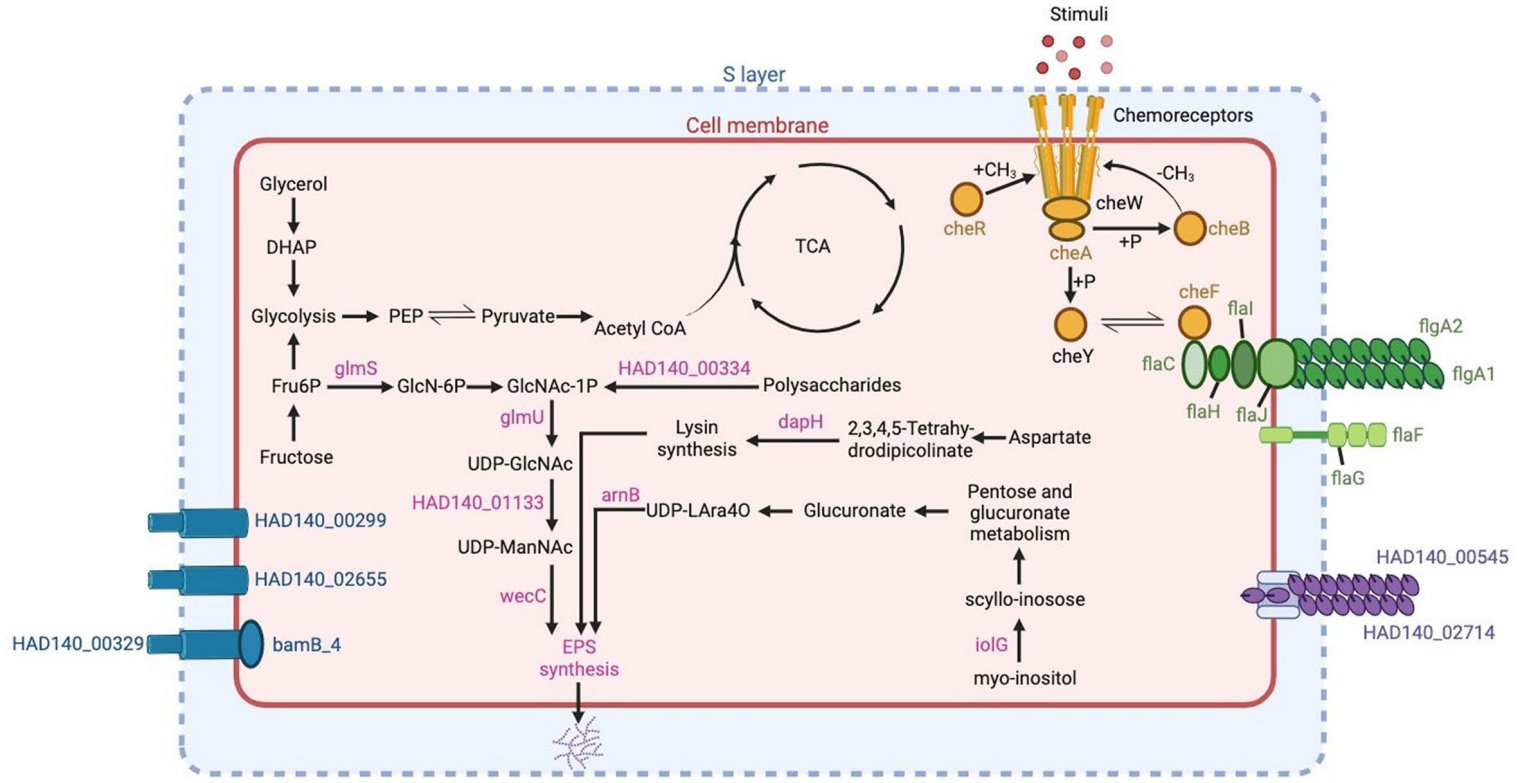
Genes related to biofilm production in *Halorubrum* sp. AD140 metabolic pathways. Blue: S-layer genes, pink: EPS synthesis genes, yellow: chemotaxis genes, green: archaella and flagella genes, purple: pili genes. Created with BioRender.com.

Biofilms found in hypersaline environments are found to hold archaea and bacteria thriving together. These biofilms facilitate genetic exchange, nutrient availability, and provide protection for microorganisms residing within them. The genome of *Halorubrum* sp. AD140 exhibits numerous biofilm gene homologs to *Halorubrum lacusprofundi* and *Haloferax volcanii* genomes. Among the queried genes, only HVO_2056 has no homolog in *Halorubrum* sp. AD140, which contributes to the biogenesis of the low-salt tetrasaccharide sugar withing the EPS operon (Kaminski et al., 2013). Genes involved in EPS synthesis include sugar and amino acid metabolizing enzymes such as *glmU, glmS, dapH, arnB, iolG* and *wecC.* Additionally, two genes were identified with the N-terminal domain of the type IV pili: HAD140_00545 and HAD140_02714. The presence of *che* genes, responsible for chemotaxis and regulation of archaella and flagella motility, was also observed. Among the genes related to archaella and flagella, *flaI, flaC, flaH, flaJ, flaG, flaF* and the archaellins *flgA1* and *flgA2* were identified. Furthermore, four S-layer genes involved in surface adhesion were found, as depicted in Figure 6. These genes collectively enable *Halorubrum* sp. AD140 to undergo biofilm formation, including surface adhesion, microcolony formation, and biofilm maturation. Finally, 4 S-layer genes were found, which participate in surface adhesion, indicated in blue in Figure 6. All these genes interact so that *Halorubrum* sp. AD140 can perform the process of biofilm formation, from surface adhesion to microcolony formation and biofilm maturation.

The genes homologs identified in *Marinococcus luteus* were based on previous annotations from different *Halomonas* and *Marinobacter* species. As expected, *Marinococcus luteus* does not have genes related to S-layer, and archaellin which are inner characteristics of Archaea domain. However, compared to *Halorubrum* sp. AD140, it contains ~64% more EPS biosynthesis genes, subdivided in nucleotide sugar synthesis, *kps* system, and ATP-binding cassette (transporters) genes, 74% more in chemotaxis and quorum sensing and ~ 80% more genes related to flagella. *Marinococcus* also possess similar genes related to flagella, although the operon component arrangement is different from the archaeon. Unlike *Halorubrum*, *Marinococcus* has five genes responsible for biofilm regulation, *csrA*, *cpdA*, *uvrY* and *wspA*. Detailed description of the prokka annotation followed by BLAST search performed to identify biofilm genes in *Halorubrum* sp. and *Marinococcus lutes* are found on Supplementary Tables 6, 7, respectively. Evaluation of biofilm formation of the AD140 co-culture was performed (Figure 3B), observing a continuous growth over time of an extracellular matrix at the bottom of the tube in static culture. Moreover, we observed biofilm formation and natural aggregation and attachment to the tube wall while the culture grows with agitation, visible at day 20.

### Metabolic network analysis

**Figure 7:**
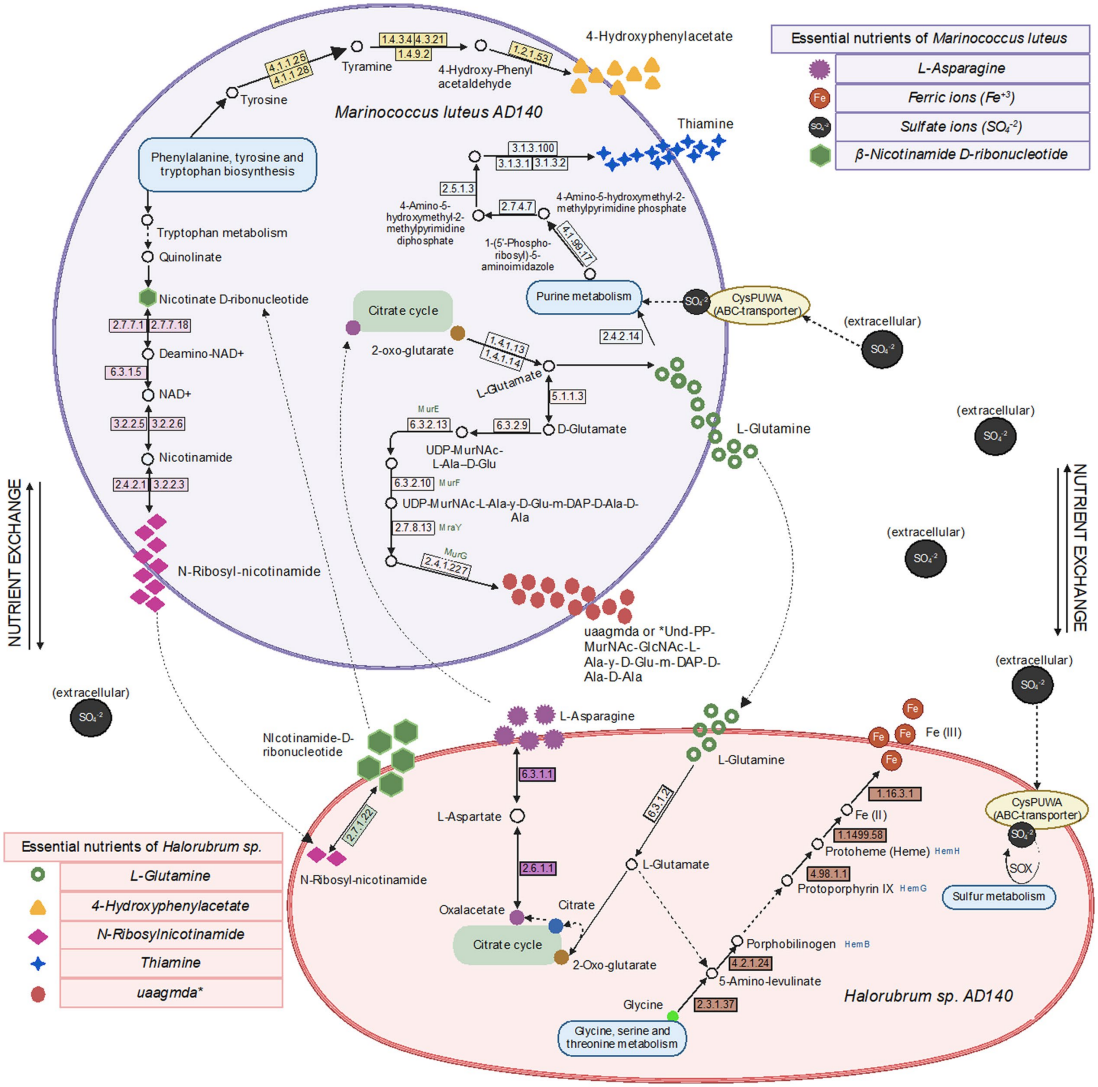
Metabolic pathways, metabolite exchange and essential nutrients between *Halorubrum* sp. and *Marinococcus luteus* model predicted using drop-out flux balance analysis. Extracellular Sulfate ions comes from one of the components of 213 and M2 media, MgSO_4_ 7H_2_O, available in the culture environment. Created with BioRender.com.

We generated genome-scale metabolic models for each species, using the prokka-annotated genomes retrieved from maxbin. Since we do not know any defined media upon which either of these strains can grow independently, we did not gap-fill beyond the basic requirement that growth must be possible when all environmental metabolites the model can uptake are present, including the composition of the 213 and M2 media. We first used these models to predict essential nutrients for both species. As expected, each requires many of the same basic ions as Cl, K, Ca_2_, Mg_2_, Mn_2_, Cobalt_2_, Zn_2_, Cu_2_. Beyond these, there are differences in nutrient requirements. The models predict that *Halorubrum* and *Marinococcus* are each auxotrophic for different amino acids (glutamine and asparagine, respectively), that *Marinococcus* requires nicotinamide from the environment, and *Halorubrum* requires multiple environmental nutrients, including thiamin. Nonetheless, in the two functional annotations (prokka and RAST) of each microorganism, it was found in *Halorubrum* elements from a pathway to synthesize nicotinamide-D-ribonucleotide which is used by the bacteria as a precursor for N-ribosyl-nicotinamide synthesis. Conversely, *Marinococcus*, synthetizes thiamin monophosphate by coupling two components thiazole and pyrimidine in separate pathways, and becoming active as thiamin diphosphate with a thiamine phosphate kinase (Figure 7).

**Figure 8:**
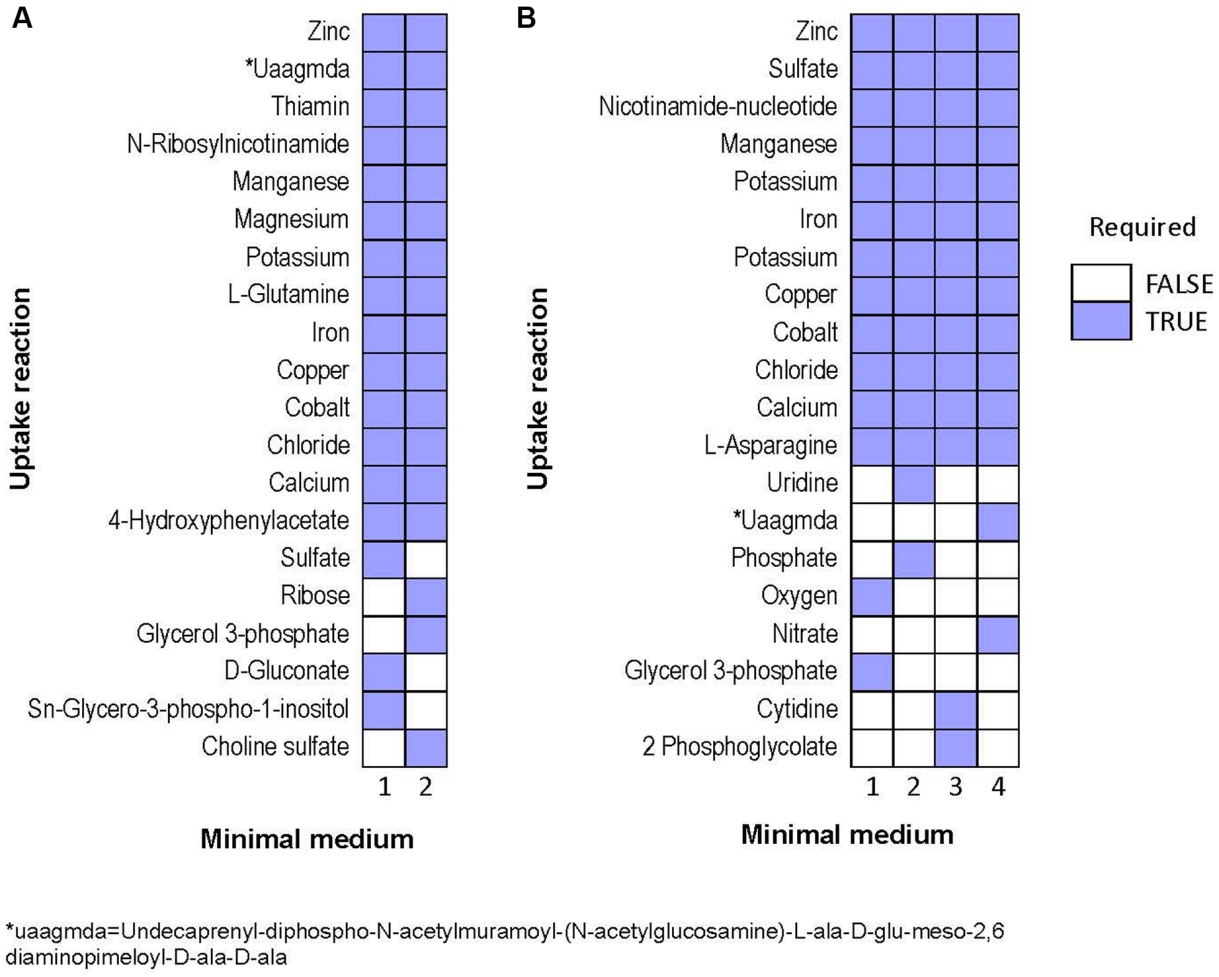
Alternative minimal media identified for **(A)** *Halorubrum* sp. and **(B)** *Marinococcus luteus*. Each column is a different set of nutrients which allow growth in flux balance analysis.

Next, we predicted potential minimal media for each species, specifically aiming at minimizing the number of compounds, rather than minimizing the summed uptake of the compounds. Over 1e6 iterations, the algorithm identified only two alternative minimal media for *Halorubrum*, and four for *Marinococcus. Halorubrum* alternative minimal media were composed of differed minimal ways to uptake sugar (ribose or gluconate), phosphate (Sn-Glycero-3-phospho-1-inositol or glycerol-3-phosphate), and sulfate (sulfate or choline sulfate). *Marinococcus’* alternative media were also composed in part of different ways to uptake an energy source, phosphate, and in one case, oxygen (Figure 8).

## Discussion

Syntrophic interactions are central to microbial communities and were foundational in the history of complex life. Despite this centrality in microbial ecology and evolution, the initial evolution and genomic basis of cross-feeding are challenging to determine. The vast majority of microorganisms cannot be cultured, limiting experimental investigation on most syntrophic interactions. And even for mixed species microbial cultures involving syntrophic interactions, determining shared substrates is daunting as microbes alter their environments in numerous diverse ways. Using a genomic approach through Next Generation Sequencing (NGS) technologies, we assessed the community context for growth of a microbial species pair. Obtained as a single isolate, neither species can be cultivated alone. When grown together in nutrient rich media, the culture reaches a high density in liquid and shows abundant growth on plates. A metabolic network analysis suggests that most auxotrophies in one species could be complemented by crossfeeding from the other species, but not all. This mix of auxotrophies and syntrophies suggests that the source microbial community contains a web of diffuse beneficial syntrophic interactions. The complexity of source microbial community is also reflected in other aspects of their genomes, including genes and genomic islands associated with secondary metabolite production, cell attachment, and horizontal gene transfer. These results suggest that the structure of microbial communities involves numerous competitive and mutualistic interactions, providing opportunities for beneficial symbioses to evolve.

Comparing the genomes of symbiotic organisms by identifying conserved genes or specific adaptations unique to symbiotic partners, along with metabolic network predictions, can aid in addressing the remaining questions about symbiotic interactions like: “How do organisms in symbiotic relationships recognize and select suitable partners?” “What are the molecular mechanisms and signaling pathways involved in the recognition and establishment of symbiotic associations?” What are the evolutionary origins of symbiotic relationships? How did these complex interactions among organisms arise, and how have they evolved? Implementing these opens a new perspective into long-standing evolutionary questions about spatio-temporal microbial symbiosis and how cross-feeding affects diversity and speciation.

### Syntrophic evidence for an early-stage symbiosis

Cultures of halophilic archaea and bacteria, like *Halorubrum* and *Marinococcus*, can be challenging to cultivate in laboratory settings. In this work, we proposed this syntrophic relation between *Halorubrum* sp. and *Marinococcus luteus* as an early-stage symbiosis due to their consistent co-occurrence in laboratory conditions, evolving to thrive as consortia, contributing to the community’s resilience and adaptability to harsh salinity conditions. One of the central questions in this work was whether these species engage in an obligate mutualistic relationship. To address their close relationship, we performed metabolic network modeling. It was found that each species had unique nutrient requirements. Importantly, most of these nutrient requirements could be produced by other species, though that was not true of all *Halorubrum* requirements. In the model, *Marinococcus* could not produce 4-hydroxyphenylacetate or thiamine. However, functional annotations coupled with media composition use to gap-fill the network, shows that *Marinococcus*, has the complete pathway to synthetize thiamine and 4-hydroxyphenylacetate. Furthermore, while both species had the capacity to produce the amino acid that the other species was auxotrophic for, this production itself depended upon the environmental availability of an amino acid that the producer itself was auxotrophic for. For instance, *Marinococcus* could only produce glutamine when provided with asparagine, and conversely, *Halorubrum* relied on asparagine to produce glutamine. This cooperative metabolic interaction can lead to increased growth rates, improved resource utilization, expanding ecological niches for the symbiotic microorganisms. Moreover, the prediction of the essential nutrients alone, suggests either the archaea or the bacteria requirements are partially fulfilled by the other, this representation might be attributed to poor annotation in archaeal references available. Archaea are distinguished by the existence of distinctive, modified versions of conventional pathways like the Entner-Doudoroff (ED), Embden-Meyerhof-Parnas (EMP), and novel pentose-degrading and CO_2_-fixing pathways catalyzed by new enzymes (Sato and Atomi, 2011; Bräsen et al., 2014).

Interestingly, the number of alternative media predicted to allow optimal growth is higher in *M. luteus* than the archaeon; given the genome plasticity, and the higher number of genes within the metabolic subsystems (Figure 4), *M. luteus* is probably more efficient utilizing nutrients present in the media.

Next, we asked whether each model could produce the compounds which the other is not capable of producing, by optimizing a sink reaction for that compound (see methods). In the model, we found that *Marinococcus* could produce L-Glutamate (gln__L_c), Undecaprenyl-diphospho-N-acetylmuramoyl-N-acetylglucosamine-L-ala-D-glu-meso-2,6-diaminopimeloyl-D-ala-D-ala (uaagmda_c) and N-Ribosylnicotinamide (rnam_c), but not 4 hydroxyphenylacetate (4hphac_c), or Thiamin (thm_c), however, the genome annotation show the presence of the elements needed for the pathway. Moreover, it could only produce as much N-Ribosylnicotinamide (rnam_c) as it was supplied with (nmn_e), potentially suggesting that this precursor is limiting to both species. On the other hand, *Halorubrum* can produce both L-Asparagine (asn__L_c) and N1-methylnicotinamide (nmn_e) during growth in a minimal medium. However, it could only produce as much N1-methylnicotinamide (nmn_e) as it was supplied with N-Ribosylnicotinamide (rnam_e).

### Other mechanisms and processes facilitating interdependence

The shift from a free-living lifestyle to symbiotic interaction is occasionally facilitated by horizontal gene transfer (HGT) events, which frequently carry traits that enable the rapid exploitation of the environment (Madsen et al., 2012; Drew et al., 2021). Archaea and Bacteria are usually found in mixed population biofilms, offering nutrient supply, protection, and boosting genetic variation through HGT events (Fröls, 2013). HGT processes can be involved in sympatric speciation and in the high rate of genetic recombination on halophilic communities (Ram Mohan et al., 2014). We found genomic evidence of biofilm production in both strains, since there are some gene homologs that participate in chemotaxis, EPS production, pili, and flagella.

The initial recognition between the two strains can involve surface interactions through adhesins or pili-like appendages that facilitate attachment to each other or a common surface. These genes were identified in *Halorubrum*, with the N-terminal domain of the type IV pili from six adhesion pili *PilA1-6*. Other studies have demonstrated that the expression of *PilA1*, *PilA2*, or *PilA6* allows the microcolony formation, while *Pil3* or *Pil4* enhance surface adhesion (Esquivel et al., 2016). Additionally, archaellum genes were identified, along with *FlaF* and *FlaG* gene-complex, which is required for archaellum assembly and motility, plus four accessory motor components, *FlaC, FlaH, FlaI*, and *FlaJ* (Shahapure et al., 2014; Tsai et al., 2020). As for *Marinococcus*, two motility-related gene clusters *fliEFGHIJKLMNOPQR and flgBCDEFGHIJKL* were identified, along with their own regulatory systems. Moreover, similar *Che* gene sets were present, *CheW* and CheA led to the activation of *CheY* gene, which acts upon *MotAB* a flagellar motor complex, in charge of the rotation of bacterial flagella through conformational alterations (Liu and Ochman, 2007; Athmika et al., 2021).

Interestingly, several biofilm genes found in both strains have more than one copy, this duplication of the genome content offers an abundance of genetic material for evolution and is suggested to be crucial in adapting to novel environments and promoting diversification (Xu et al., 2020). Meanwhile, some genes belonging to operon-complexes were not found, probably due to adaptive evolution trough gene loss, as a rapid response to environmental fluctuations.

Genomic islands are also part of those exchanged elements, and another example of their interaction capacity is observed in the genome content of *Halorubrum* sp. and *M. luteus*, with accessory functions of symbiosis, secondary metabolites production, and antibiotic resistance (see Supplementary Tables 3, 4). Particularly, *Halorubrum* comprises seven genomic islands related to symbiosis, while *Marinococcus* possesses four. According to the metabolic prediction analysis, thiamine constitutes one of the essential nutrients for the *Halorubrum* strain. Noteworthy, it was identified the presence of a set of islands with thiamine and sugar catabolism capacity, allowing the archaea to acquire and metabolize energy from compounds found in the environment (Lacour et al., 2006).

One of the singularities, is the presence of arsenic and cadmium resistant genes in both, *Halorubrum* sp. (island 19) and *Marinococcus* sp. (island 3). Previous studies have observed the abundance of arsenic and cadmium in the CCB (Valdespino-Castillo et al., 2018; García-Ulloa et al., 2022).

Arsenic resistance genomic islands have been characterized particularly in microbes that inhabit oligotrophic environments. Phosphate and arsenate are chemical analogs, substituting each other in chemical reactions (Strawn, 2018). The arsenic metabolism genes can help the microorganisms to use low concentrations of phosphorus under high arsenic concentrations (Li et al., 2013). The scarcity of phosphorus can reinforce arsenic uptake, while high concentrations impede arsenic uptake (Strawn, 2018). Oligotrophic conditions are a main characteristic of the Cuatro Cienegas Basin environment (Souza et al., 2008), strongly marked by a phosphorus limitation. Therefore, the evidence of genomic islands related to arsenic metabolism, might indicate an alternative way of *Halorubrum* sp. to survive in the CCB environment by up taking the arsenic available. Interestingly, both cadmium resistance and arsenic resistance often co-occur (Parsons et al., 2020).

### Convergent evolution to an extreme environment

The presence of the highly genetically divergent species *Halorubrum* sp., and *M. luteus* in CCB suggests that both microorganisms have acquired similar traits, using unique metabolic pathways and osmoregulation mechanisms, allowing them to thrive in high salt concentrations and fluctuating environments through convergent evolution events by evolving specialized transporters, ion channels, or regulatory systems. The Cuatro Cienegas Basin (CCB) has often shown the surprising presence of microorganisms limited to certain distinct environments, and the extant marine environment, along with its community, has been preserved (Souza et al., 2006, 2018). However, given the ancestral marine state of CCB, we might be able to explain the presence of some unexpected taxa, such as *M. luteus*, commonly found in salt lakes (Wang et al., 2009; Fox-Powell and Cockell, 2018). The functional annotation of both strains revealed the presence of an osmotic regulation system composed of elements coding for compatible solutes such as trehalose, glycerol, glutamate synthesis. These solutes help maintain cell turgor to counteract osmotic stress. Independently, *Halorubrum* sp. has a complete *Trk* gene system in charge of potassium uptake, meanwhile, *M. luteus* has ectoine and betaine biosynthesis, as well as choline uptake regulation pathways, regulated by *gbuA*, *gbuB*, *gbuC* and *nsmX* genes.

While identifying adaptative events can be challenging, the independent evolution of similar traits in multiple lineages is one of the strongest indications that adaptation has occurred. Despite the challenges and limitations of identifying adaptive events, this criterion can help us to understand better the mechanisms driving biological evolution and the relationship between microorganisms and their environment. Part of this suggested evidence is the presence of terpene biosynthetic clusters in both, *Halorubrum* and *Marinococcus* strains. This cluster is distinctive for each one; although both clusters have a phytoene synthase gene, the gene content and gene arrangement is different. Phytoene synthase gene is conserved in all terpene and carotenoid-producing archaea and in most carotenoid-producing bacteria. However, carotenoid and terpene biosynthesis pathways are more diverse in bacteria. The study of secondary metabolites in archaea is still rare, and most archaea only have one or two BGCs, usually a terpene or a bacteriocin BGC (Wang et al., 2019). Most of the genes in bacteria associated with terpene biosynthesis belong to the *crt* gene family (Sandmann, 2021). This correlates with the observed genes in the terpene BGC in *M. luteus*, as most genes in this cluster belong to the *crt* family. The terpene biosynthesis is directly involved in the carotenoid biosynthesis pathways, and carotenoid production is common in halophilic archaea and bacteria, as one of its functions stabilizes the cell membrane (Sandmann, 2021).

Perhaps surprisingly, we also observed little, or no, evidence of CRISPR-Cas elements. No CRISPR elements were observed in the *Marinococcus* genome. And in the *Halorubrum* genome, we have only a low evidence level, which can be sequencing artifacts (see Supplementary Table 5). Although the incidence of CRISPR-Cas elements in archaea is high, it has been reported some *Halorubrum* archaea do not have them. This might be due to the deterioration of CRISPR arrangements in the genome, which can occur during DNA repair processes or because the need for the system is not constant (Fullmer et al., 2014). Likewise, the low concentrations of phosphorus present in the CCB can limit the plasticity of the genome, in addition to the fact that the transformation is reduced under these conditions (Souza et al., 2008).

## Conclusion

The comparative genomic analysis revealed intriguing similarities and differences between the halophilic *Halorubrum* sp. and *M. luteus*. Both organisms exhibit convergence in terms of the genetic components involved in their adaptation to high-salt environments. The shared presence of gene clusters related to osmo-adaptation and ion transport suggests convergent evolution in response to salinity. However, the presence of distinct genomic elements highlights the influence of their respective evolutionary histories. Along with the metabolic prediction, the data suggest that the two species partially complement each other’s metabolism, as well as greater complexity and environmental dependency than a simple, direct cross-feeding arrangement. Noteworthy, other organisms thriving in the same niche, such as bacteria, archaea, fungi, and protozoa, may also improve the survival, fitness, and metabolism of these strains. Moreover, models derived from metabolic network analysis improve understanding of the ecological complexity and dynamics within microbial communities, offering new insights into metabolism and novel culture-media alternatives. Future work to delve into the dynamic behavior of the co-culture is needed. To increase experimental tractability, time-lapse microscopy can enable us to visualize the physical interactions between archaea and bacteria, providing insights into spatial arrangements and cellular associations. Moreover, conducting stable isotope labeling techniques will help trace the flow of specific nutrients and the transfer of resources.

## Data availability statement

Complete genome data is available at NCBI, BioProject PRJNA1003106.

## Author contributions

NM-C: Conceptualization, Formal analysis, Investigation, Methodology, Project administration, Software, Supervision, Validation, Visualization, Writing – original draft, Writing – review & editing. AT-C: Data curation, Formal analysis, Investigation, Methodology, Software, Visualization, Writing – review & editing. JC: Methodology, Validation, Writing – review & editing, Data curation, Formal analysis, Investigation, Software, Visualization. WH: Supervision, Validation, Visualization, Writing – review & editing. ST-Z: Methodology, Validation, Project administration, Supervision, Writing – review & editing. MT: Supervision, Validation, Visualization, Writing – review & editing, Conceptualization, Funding acquisition, Project administration, Resources, Writing – original draft.
